# Applying the Maternal Near Miss Approach for the Evaluation of Quality of Obstetric Care: A Worked Example from a Multicenter Surveillance Study

**DOI:** 10.1155/2014/989815

**Published:** 2014-07-24

**Authors:** Samira Maerrawi Haddad, Jose Guilherme Cecatti, Joao Paulo Souza, Maria Helena Sousa, Mary Angela Parpinelli, Maria Laura Costa, Rodolfo C. Pacagnella, Ione R. Brum, Olímpio B. Moraes Filho, Francisco E. Feitosa, Carlos A. Menezes, Everardo M. Guanabara, Joaquim L. Moreira, Frederico A. Peret, Luiza E. Schmaltz, Leila Katz, Antonio C. Barbosa Lima, Melania M. Amorim, Marilia G. Martins, Denis J. Nascimento, Cláudio S. Paiva, Roger D. Rohloff, Sergio M. Costa, Adriana G. Luz, Gustavo Lobato, Eduardo Cordioli, Jose C. Peraçoli, Nelson L. Maia Filho, Silvana M. Quintana, Fátima A. Lotufo, Carla B. Andreucci, Márcia M. Aquino, Rosiane Mattar

**Affiliations:** ^1^Department of Obstetrics and Gynaecology, School of Medical Sciences, University of Campinas (UNICAMP), R. Alexander Fleming 101, P.O. Box 6030, 13083-881 Campinas, SP, Brazil; ^2^Centre for Research on Reproductive Health of Campinas (Cemicamp), R. Vital Brasil 200, 13083-888 Campinas, SP, Brazil; ^3^Federal University of Amazonas, Manaus, AM, Brazil; ^4^CISAM, School of Medical Sciences, Recife, PE, Brazil; ^5^Federal University of Ceará, Fortaleza, CE, Brazil; ^6^Federal University of Bahia, Salvador, BA, Brazil; ^7^Hospital Geral Cesar Cals, Fortaleza, CE, Brazil; ^8^Hospital Geral de Fortaleza, Fortaleza, CE, Brazil; ^9^Maternidade Odete Valadares, Belo Horizonte, MG, Brazil; ^10^Hospital Materno Infantil, Goiania, GO, Brazil; ^11^IMIP, Recife, PE, Brazil; ^12^Federal University of Pernambuco, Recife, PE, Brazil; ^13^Federal University of Campina Grande, PB, Brazil; ^14^Federal University of Maranhão, São Luis, MA, Brazil; ^15^Federal University of Paraná, Curitiba, PR, Brazil; ^16^Federal University of Paraíba, João Pessoa, PB, Brazil; ^17^Hospital Maternidade Fernando Magalhães, Rio de Janeiro, RJ, Brazil; ^18^Federal University of Rio Grande do Sul, Porto Alegre, RS, Brazil; ^19^Hospital Maternidade Celso Pierro, Campinas, SP, Brazil; ^20^Instituto Fernandes Figueira, Rio de Janeiro, RJ, Brazil; ^21^Hospital Israelita Albert Einstein, Sao Paulo, SP, Brazil; ^22^University of the State of São Paulo, Botucatu, SP, Brazil; ^23^Jundiaí School of Medicine, Jundiaí, SP, Brazil; ^24^University of São Paulo, Ribeirão Preto, SP, Brazil; ^25^Santa Casa de Limeira, Limeira, SP, Brazil; ^26^Santa Casa de São Carlos, SP, Brazil; ^27^Maternidade Leonor Mendes de Barros, Sao Paulo, SP, Brazil; ^28^Federal University of São Paulo, SP, Brazil

## Abstract

*Objective*. To assess quality of care of women with severe maternal morbidity and to identify associated factors. *Method*. This is a national multicenter cross-sectional study performing surveillance for severe maternal morbidity, using the World Health Organization criteria. The expected number of maternal deaths was calculated with the maternal severity index (MSI) based on the severity of complication, and the standardized mortality ratio (SMR) for each center was estimated. Analyses on the adequacy of care were performed. *Results*. 17 hospitals were classified as providing adequate and 10 as nonadequate care. Besides almost twofold increase in maternal mortality ratio, the main factors associated with nonadequate performance were geographic difficulty in accessing health services (*P* < 0.001), delays related to quality of medical care (*P* = 0.012), absence of blood derivatives (*P* = 0.013), difficulties of communication between health services (*P* = 0.004), and any delay during the whole process (*P* = 0.039). *Conclusions*. This is an example of how evaluation of the performance of health services is possible, using a benchmarking tool specific to Obstetrics. In this study the MSI was a useful tool for identifying differences in maternal mortality ratios and factors associated with nonadequate performance of care.

## 1. Introduction

The outcome of a critically ill patient is a result of clinical and individual factors, including previous health status, physiologic reserve, disease diagnosis, and also adequacy of care provided during the disease. Thus, it is difficult to individually analyze and predict morbidity and mortality outcomes in critically ill patients [1]. Stratification of patient groups according to clinical severity may facilitate interpretation of these results by comparing similar groups [2].

Some scoring systems are capable of quantifying severity, for example, the APACHE (Acute Physiology and Chronic Health Evaluation), SAPS (Simplified Acute Physiology Score), SOFA (Sequential Organ Failure Assessment), and MPM (Mortality Prediction Model) [1]. However, they were developed using general populations of critically ill patients in high income countries. Considering severe pregnancy complications, several factors seem to reduce the capacity to classify severity and predict mortality among pregnant women with these models.

The different physiologic parameters, diseases unique to pregnancy, and a population largely composed of young women who were previously healthy contribute to the little applicability of these tools in Obstetrics [3]. As a result, traditional risk stratification models usually overestimate mortality among pregnant women, which may hinder analysis of the performance of care provided and interpretation of morbidity and mortality outcomes [3].

“Benchmarking” may be understood as a reference point against which comparisons can be made, regarding the performance between facilities and/or best practice. The demand for this type of data is growing, not only due to initiatives to pay for performance but also because of clinical, administrative, and research applications. Performance feedback at an institutional or individual level may lead to an improvement in overall performance [2].

Several initiatives for maternal and infant health have been implemented worldwide, aimed at achieving the millennium development goals (MDG) [4–8]. Nevertheless, advances made over the years are below those required for effective morbidity and mortality reduction. Structured health systems are identified as fundamental to obtain better results and accelerate progress for achieving these goals [9, 10].

The World Health Organization (WHO) used organ dysfunction criteria and parameters of extreme severity specific to Obstetrics to define life-threatening conditions associated with pregnancy, standardizing the maternal near miss criteria [8]. A maternal near miss is an event in which a woman nearly died, but survived a severe complication occurring during pregnancy, childbirth, or within 42 days of its termination. It represents the extreme degree of organ dysfunction/failure in the wide spectrum of morbidity and differs from death only by the final outcome [8].

Until this definition, several studies used different parameters for severe morbidity, such as admission to intensive care units (ICU) or clinical diagnoses [11–14]. The first retrospective validation of these criteria was performed in a population of obstetric patients admitted to ICU, using the total maximum SOFA score as the gold standard and showing that the WHO near miss criteria obtained a sensitivity and specificity of 99.2 and 86.0%, respectively, for the identification of organ failure in at least one organ system [15].

The Brazilian Network for Surveillance of Severe Maternal Morbidity was a prospective study aimed at identifying potentially life-threatening and maternal near miss cases [16, 17]. Assuming that a woman suffering from a near miss event is exactly like one who has died, except for the outcome, criteria would be validated if all maternal deaths were identified and if the false-positive cases represented exactly the near miss cases. The performance of the WHO near miss criteria was confirmed, with a sensitivity and specificity of 100 and 92%, respectively [18]. In this study a tool called maternal severity index (MSI) was also developed specifically to predict mortality for the obstetric population. This appears to be a first step in making a case-mix analysis and a comparison between obstetric services by matching similar populations [18].

Strategies aimed at strengthening health systems are necessary. However, many systems still do not have the capacity to measure and understand their own weaknesses, making it difficult for healthcare policy managers to incorporate scientific strategies towards strengthening systems [10]. The maternal near miss approach may be a tool for assessment of quality of maternal care provided. As a result, standardization and comparison can be made between maternal morbidity groups from different locations and over time, identifying weaknesses [7]. Thus, the aim of this study was to simulate the evaluation of an obstetric health system, through analysis of the performance of care in the Brazilian Network for the Surveillance of Severe Maternal Morbidity, using the maternal near miss criteria approach.

## 2. Materials and Methods

### 2.1. Ethics Statement

Research protocol was approved by the Institutional Review Board of the coordinating institution (University of Campinas) on 5 May 2009 (Document CEP 027/2009). The study was approved by the local Institutional Review Board of each participating center and also nationally. Each center was previously consulted regarding this analysis of performance and data publication. Approval was unanimous. To ensure confidentiality, each center is not identified and received confidential information on the category it was classified in order to be able to adopt procedures to improve quality of care provided.

### 2.2. Study Population

The Brazilian Network for Surveillance of Severe Maternal Morbidity was a cross-sectional multicenter study aimed at identifying severe maternal morbidity cases, using the new WHO definition [8, 16]. From July 2009 to June 2010, 27 referral hospitals, representing a purposeful sample of the Brazilian health facilities caring for women's deliveries, made a prospective surveillance to identify severe maternal morbidity/near miss cases.

The study was planned in detail, with preparatory meetings to discuss methods and procedures with participants from all centers. In addition to personal and clinical information of each case of severe maternal morbidity identified, a rigorous system of screening for any of the three delays in obstetrical care was also implemented [19]. After prospective data collection was completed, a rigorous checking system for data consistency was developed. Additional details on method and procedures are in other publications [16, 17].

### 2.3. Development of a Model for Mortality Prediction

Previously, it was possible to build a model for mortality prediction, named maternal severity index (MSI) [18]. Briefly, two models for mortality prediction were developed. First, it was confirmed that the number of near miss markers could be related to mortality and this correlation was called maternal severity score (MSS). The WHO near miss markers are shown in Table 1, distributed as Group A (organ dysfunction) and Group B (severe dysfunction/failure). Two models of  bivariate logistic regression were then developed and tested to describe the relationship between severe morbidity and mortality. For this, the total study population was divided into two subpopulations “A” and “B” to develop and test the prediction model, respectively. The sample size of population “B” was obtained considering a probability of 0.05 for type I error, 0.20 for type II error, and a minimum area under the ROC (receiver operating characteristic) curve of 0.80.

**Figure 1:**
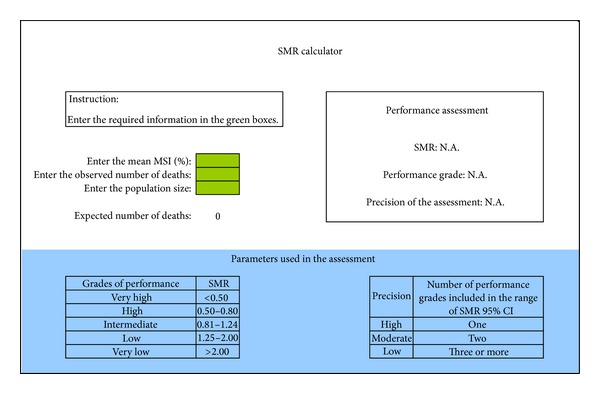
SMR calculator.

The first model was a univariate analysis including only MSS or the number of severity markers. The second used univariate analyses considering MSS, distal predictors of mortality (such as demographic and obstetric characteristics), and near miss criteria as independent variables and the outcome maternal death as the dependent variable. Variables significantly correlated with mortality were selected for multivariate analysis. Positive or negative correlation coefficients (*β*) were attributed to each variable included in the model. Calibration and discrimination of models were performed. Model 2 showed the best performance for mortality prediction. Therefore, it was chosen to be the maternal severity index (MSI) [18]. Briefly, the SMI is estimated through an equation which takes into account the MSS (number of life-threatening conditions) and the presence of some associated specific conditions (life-threatening condition identified in the first 24 hours of hospital stay, severe preeclampsia, cancer, any marker of cardiovascular failure, any marker of respiratory failure, and hysterectomy), which are the variables significantly correlated with mortality mentioned above. With the SMI thus estimated for each case and the mean SMI for any specified group, the number of expected deaths can be determined. When the number of observed deaths is compared with the number of expected deaths, the concept of standardized mortality ratio (SMR) is used. To simplify the estimation of SMR, a calculator was developed (Figure 1).

### 2.4. Analysis of Performance of Network Centers

The mean MSI for each center was obtained and the standardized mortality ratio (SMR) was calculated for each one (Figure 1). SMR is the ratio between observed mortality in the population and expected mortality by mortality prediction based on severity of the case expressed by MSI. To allow calculation of SMR for all centers, a value of 0.1 was attributed, when no death was observed or there was no expected death due to the small sample size and/or low complexity of cases in that center.

In the original model, the categorization of performance was based on cutoff points selected assuming the normal distribution into five classes of care and the understanding that SMR < 0.5; that is, the occurrence of half or less than the expected number of deaths could correspond to excellent care. Thus, the five categories of performance were defined as very high, high, intermediate, very low, and low [18].

In this analysis, SMR was calculated from the mean MSI for each of the 27 study centers. Due to the relatively small number of centers and to make analyses more consistent as an exercise to evaluate large systems, the original classification was modified, and groups were relocated to two new categories. Categories “very high,” “high,” and “intermediate,” including SMR < 0.5 to 1.24, were reclassified as “adequate” care. Categories “low” and “very low,” with SMR of 1.25 to >2, were recoded as “nonadequate.” Thus, this performance was analyzed as a dependent variable. Using both groups of adequacy of care, variables related to structure, process, management, and delays were correlated as independent variables. Although SMR was calculated for each health facility, it should not be understood as an evaluation only of the care the hospital provided but the general care those women received. This includes not only the hospital activities/responsibilities but also those from the woman herself, her family, community, and access to health services.

Furthermore, patient outcomes, their respective indicators, and main causes of complication were evaluated for both groups of level of performance. Finally, Poisson multiple regression analysis was performed, using the level of performance as a dependent variable, estimating the prevalence ratio and its respective 95% CI to identify variables independently associated with performance. A 5% statistical significance level was used. All measures of effect in the study design and their respective *P* values were calculated after adjusting for cluster effect.

## 3. Results

In this period, when these facilities took care of 82,388 deliveries with 82,144 live births (representing a fraction of around 2.8% of all annual live births in the country), 9555 women had severe pregnancy complications, with 770 near miss cases and 140 maternal deaths. In Table 2, the mean MSI, number of observed deaths, sample size (potentially life-threatening and near miss conditions), expected number of deaths, SMR, and, finally, the recategorized level of performance of  care are presented for each of  the 27 centers, 17 being classified as having “adequate” and 10 as “nonadequate” performance. Generally, the overall performance of Network was adequate, since there were only two more maternal deaths than expected due to severity of cases (SMR = 1.02).

Table 3 shows the distribution of outcomes of severity and of main causes of morbidity, according to the level of performance of the centers. Although differences were not significant, there was proportionally almost twice the proportion of deaths among centers classified as “nonadequate” care. Maternal health indicators also show that the occurrence of maternal near miss was similar between performance groups, but there was an almost twofold increase in the maternal mortality ratio (MMR) in the group with nonadequate care.

Structure indicators according to level of performance are shown in Table 4. Centers with “adequate” performance were located with an almost 2.5 higher prevalence in the southeast and south of the country, but this difference was not significant. When process and management indicators (Table 5) were analyzed, there was twofold increase in ICU admissions in adequate care centers, but this difference was also not significant.

Association between delay in care and the level of performance is shown in Table 6. Generally, both detection of any type of delay and some specific categories of delays, particularly those concerning the third delay (related to quality of care), were significantly more common in the nonadequate performance group. Considering all the predictive variables included in the multiple regression analysis (Table 7), the use of magnesium sulfate and location of the health center in the south or southeast were the main variables independently associated with adequate performance of care.

## 4. Discussion

With the use of the MSI and SMR, it was possible to assess the performance of centers from the Brazilian Network for Surveillance of Severe Maternal Morbidity. This was a first initiative to use the near miss concept as a tool for assessment of case-mix and adequacy of care received in Obstetrics. However, as already stated, this assessment of the quality of obstetrical care is probably more appropriate for a specific population that “receives” the care than for a specific health facility that “provides” the care. This is because the global assessment does not imply only the activities performed at the hospital but also the characteristics of women and their community services and access to health services. If a woman arrives late at the hospital in a very severe almost dying condition, she is more likely to die even if the hospital is tertiary, well equipped, and with a good trained staff. The responsibility for such a death cannot be attributed solely to this hospital. In other words, the care provided by the hospital can be adequate while the care received by the woman can be not adequate.

In the classification of performance, sample size per center corresponds to the number of cases with potentially life-threatening conditions and threatening life conditions (near miss and maternal death) and not the total number of live births, because only women presenting with some severity indicators were included in the study. Although calculation of SMR for all centers was possible after attributing a value of 0.1 to those that did not present any observed or expected death, these estimates had low accuracy. Therefore, the MSI seems limited and less precise for use in populations with a small number of cases, with lower clinical severity (low MSI) and those with no observed death. The lower accuracy of estimates in these cases should increase attention and care for interpreting the performance of these health services.

Centers participating in this Brazilian Network were selected considering their availability to participate in the study, the total number of annual deliveries, and geographic location. Thus, the estimated sample size could be achieved, ensuring a broad distribution in national territory. Nevertheless, most of these hospitals were linked to large university institutions or had teaching activities, and this implies that they are mostly referral for healthcare of severe pregnancy complications, with evidence-based protocols and similar standards of care. The relative homogeneity of these facilities may have contributed to the lack of significant differences between levels of performance of care and most variables related to the profile of severity of illness, cause of morbidity, and structure and management indicators, in addition to the limited number of centers participating in the study.

The greater proportion of infectious causes among centers with adequate performance may be perceived as better adequacy to international protocols for the management of sepsis [20], already widely known among academic medical services. Referral of these cases to hospitals equipped with a high complexity arsenal is a determining factor for the survival of these patients.

As observed, there was an almost twofold increase in proportion of deaths in centers with nonadequate performance. However, the prevalence of maternal near miss was practically the same in both groups of performance. This is in agreement with recent knowledge that severe pregnancy complications occur practically in the same frequency in all countries and regions, regardless of the level of development and availability of resources. In fact, the varying factor is mortality, which is always higher in contexts of lower development and scarce resources, as currently demonstrated [21, 22].

In this study, information on potential delays in obtaining care among women suffering severe complications was collected. In addition to objective information from medical charts, local researchers made a subjective assessment and searched for the three types of delay in obtaining care [19]. The presence of any delay in care was significantly related to a worse performance of service. These results are in agreement with the concept that the main preventive factors in decreasing maternal mortality are delays in the care process, from symptom identification by the patient to the provision of adequate treatment by healthcare professionals [19]. Globally there was a greater delay in providing services in nonadequate care centers, mainly due to the absence of blood products and difficulty in establishing communication between services and/or regulatory centers.

These findings seem to follow a presumptive logic. Subjects living in geographic regions with difficult access have the greatest difficulty in seeking medical care, including antenatal followup. Healthcare facilities close to these homes are also probably on the outskirts of large cities and high-complexity hospitals are usually in the center of these cities. In general, this peripheral healthcare equipment does not have an adequate structure to provide immediate care and monitor complications. There is also difficulty in regulating complex cases to large referral centers. Finally, successive delays in providing care are related to a higher number of severe outcomes and deaths.

This hypothesis may corroborate information that health system strengthening may actually have the greatest impact on improvement of clinical care [10]. For instance, prompt action of the whole health system for hierarchization of care, according to demand of severity, could ensure a reduction in deaths by improving adequacy of care. These strategies would go beyond the sole responsibility of the healthcare managers. Most probably, social development actions, civil, and transport infrastructure are necessary to correct determining factors for the health of these more vulnerable populations.

The MSI for mortality prediction follows the same indications and limitations of similar models, such as the APACHE and MPM. Use for individual evaluation is limited, since the greatest outcome predictor is individual response to therapy administered [2]. In addition, waiting to provide specific measures only when certain markers of severity emerge is not recommended. Individual clinical care is dynamic and the use of severity scores for decision making may delay healthcare, with poor provision of adequate resources. Most existing scores consider the clinical parameters obtained in the first 24 hours after ICU admission and do not assess time, care, or alterations present before it [2]. MSI was developed using data from the prehospital phase until discharge, using several sources distributed all over the country, which may increase the accuracy of its prediction capacity. However, if the development of the model and the use in the same population could be a limitation of the method, tests in other samples are needed for external validation and in fact it was already performed in a huge sample from the WHO study [22].

The SMR may be perceived as the evaluation of performance of a system rather than of a health service alone. In this study, SMR was recategorized as “adequate” and “nonadequate,” the cutoff point being the limit between intermediate and low care from the original classification [18]. Thus a group of centers that actually had SMR above 1 was classified as providing “adequate” care. Centers that could not prevent any expected death and in some cases had a slight increase in the number of deaths in relation to the expected due to severity of their cases were categorized as having adequate performance. Although this methodological option may have reduced the identification of variables related to actual excellent care (SMR between 0 and 0.8), it was a strategy adopted as a form of simplifying data analysis and prioritizing identification of centers with nonadequate performance. In the future, with a larger number of participating hospitals and subjects, it is likely that analysis with three categories (e.g., high, intermediate, and low performance) may be a valuable strategy for evaluating all the components of performance individually.

## 5. Conclusions

In the Brazilian Network for Surveillance of Severe Maternal Morbidity, the near miss approach was used to simulate analysis of an obstetric health system. After applying the MSI and SMR, analysis of the performance of services received was possible and its associated factors were assessed. Problems arising from the health system organization were identified as significant, especially those related to accessibility to health services and quality of medical care provided.

The use of this specific tool for mortality prediction may contribute to the analysis of obstetric health systems and identification of weaknesses. Furthermore, it may help to strengthen these systems, with an effective reduction in deaths. Nevertheless, new studies in different populations should be conducted for external calibration of tools developed in the Brazilian Network.

## Figures and Tables

**Table 1:** The WHO set of severity markers (life-threatening conditions) used in maternal near miss assessments.

	Group A	Group B
Cardiovascular dysfunction	Shock	pH < 7.1
	Lactate > 5	Use of continuous vasoactive drugs
		Cardiac arrest
		Cardiopulmonary resuscitation (CPR)

Respiratory dysfunction	Acute cyanosis	Gasping
	Respiratory rate >40 or <6/min	PaO_2_/FiO_2_ < 200 mmHg
	Oxygen saturation <90% for ≥60 minutes	Intubation and ventilation not related to anesthesia

Renal dysfunction	Oliguria nonresponsive to fluids or diuretics	Creatinine ≥300 mmol/L or ≥3.5 mg/dL
		Dialysis for acute renal failure

Coagulation/hematological dysfunction	Clotting failure	Acute thrombocytopenia (<50 000 platelets)
	Transfusion of ≥5 units of blood/red cells	

Hepatic dysfunction	Jaundice in the presence of preeclampsia	Bilirubin >100 mmol/L or >6.0 mg/dL

Neurological dysfunction	Metabolic coma (loss of consciousness AND the presence of glucose and keto acids in urine)	Coma/loss of consciousness lasting 12 hours or more
	Stroke	
	Status epilepticus/uncontrollable fits/total paralysis	

Uterine dysfunction	Hysterectomy due to infection or hemorrhage	

Source: reference [18].

**Table 2:** Analyses of MSI, SMR, and level of performance for each center of the Brazilian Network for Surveillance of Severe Maternal Morbidity.

Center	MSI (%)	Observed number of deaths	Sample size	Expected deaths	SMR	(95% CI)	Performance
1	0.44%	5	566	2	2.01	(0.65–4.69)	Non A
2	3.54%	4	96	3	1.18	(0.32–3.01)	A
3	0.57%	0	253	1	0.00	(0.00–2.56)	A
4	7.74%	6	112	9	0.69	(0.25–1.51)	A
5	1.92%	2	210	4	0.50	(0.06–1.79)	A
6	1.93%	17	1086	21	0.81	(0.47–1.30)	A
7	1.81%	3	172	3	0.96	(0.20–2.82)	A
8	0.25%	1	1050	3	0.38	(0.01–2.12)	A
9	1.40%	4	155	2	1.84	(0.50–4.72)	Non A
10	3.84%	30	609	23	1.28	(0.87–1.83)	Non A
11	0.68%	3	186	1	2.37	(0.49–6.93)	Non A
12	7.91%	8	98	8	1.03	(0.45–2.03)	A
13	4.55%	3	154	7	0.43	(0.09–1.25)	A
14	0.59%	0	841	5	0.00	(0.00–0.74)	A
15	3.24%	9	369	12	0.75	(0.34–1.43)	A
16	1.04%	11	945	10	1.12	(0.56–2.00)	A
17	1.15%	5	294	3	1.48	(0.48–3.45)	Non A
18	1.39%	1	66	1	1.09	(0.03–6.08)	A
19	0.69%	8	920	6	1.26	(0.54–2.48)	Non A
20	4.05%	8	118	5	1.67	(0.72–3.30)	Non A
21	0.53%	0	48	0	0.00	(0.0–14.52)	A
22	3.25%	5	74	2	2.08	(0.68–4.85)	Non A
23	0.47%	3	465	2	1.37	(0.28–4.01)	Non A
24	0.49%	1	263	1	0.78	(0.02–4.32)	A
25	1.66%	3	65	1	2.78	(0.57–8.12)	Non A
26	0.05%	0	59	0	0.00	(0.0–122.9)	A
27	0.11%	0	281	0	0.00	(0.0–11.93)	A

Overall	1.44%	140	9555	138	1.02	(0.86–1.20)	A

A: adequate; Non A: nonadequate; MSI: maternal severity index; SMR: standardized mortality ratio.

**Table 3:** Distribution of cases of severe maternal morbidity according to the group of severity, main causes of morbidity, and level of performance of care.

	Level of performance of care				*P* ^1^
	Adequate		Nonadequate		
	*n*	(%)	*n*	(%)	
Outcome of severity^&^					0.4
PLTC	5,543	(90.8)	3,102	(89.9)	
MNM	494	(8.1)	276	(8.0)	
MD	66	(1.1)	74	(2.1)	
Total	**6,103**	**(100.0)**	**3,452**	**(100.0)**	
Main causes					
Hemorrhage	1,438	(23.6)	840	(24.3)	0.92
Hypertension	4,323	(70.8)	2,383	(69.0)	0.83
Infection	83	(1.4)	17	(0.5)	*0.04 *
Clinical-surgical	614	(10.1)	409	(11.8)	0.5
Total	**6,103**		**3,452**		
Maternal health indicators					
MNMR	10/1000 LB		8.4/1000 LB		0.89
MMR	134/100.000 LB		225/100.000 LB		<*0.001 *
SMOR	11.4/1000 LB		10.6/1000 LB		0.13
LB	49.275		32.869		

LB: live births; MD: maternal death; MMR: maternal mortality ratio; MNM: maternal near miss; PLTC: potentially life-threatening condition; SMOR: severe maternal outcome ratio.

^&^Comparisons: PLTC × (NM + MD): *P* = 0.721; (PLTC + NM) × MD: *P* = 0.123.

^1^ *P* value adjusted for cluster effect.

**Table 4:** Distribution of study centers according to structure indicators and level of performance of care.

Structure indicator^@^	Level of performance		*P* ^1^
	Adequate	Nonadequate	
Type of ICU			∗
Obstetric	5 (29)	2 (20)	0.68^#^
Only general ICU	9 (53)	5 (50)	>1^#^
None	3 (18)	3 (30)	0.64^#^
Level of complexity			>1^#^
Secondary	3 (18)	2 (20)	
Tertiary	14 (82)	8 (80)	
Geographic region			0.06^#^
North, Northeast, and Center-West	5 (29)	7 (70)	
Southeast and South	12 (71)	3 (30)	
Level of government			∗
Municipal	2 (12)	2 (20)	0.61^#^
State	5 (29)	4 (40)	0.68^#^
Federal	7 (41)	3 (30)	0.69^#^
Nonpublic	3 (18)	1 (10)	>1^#^
Total	**17 (100)**	**10 (100)**	

^@^The following indicators were not taken into account: teaching hospital; blood bank; neonatal ICU and round the clock anesthetic available, due to the fact that almost all centers had these indicators.

∗Chi-square test not applicable for general comparison.

^#^Fisher Exact Test.

^1^ *P* value adjusted for cluster effect.

**Table 5:** Distribution of cases of severe maternal morbidity according to process and management indicators and level of performance of care.

Process indicators	Level of performance				*P* ^1^
	Adequate		Nonadequate		
	*n*	(%)	*n*	(%)	
Spontaneous access to facility	3.227	(54.2)	1.082	(35.7)	0.06
Total (a)	**5.958**	**(100.0)**	**3.033**	**(100.0)**	
Way pregnancy is terminated (cesarean)	3.748	(61.7)	2.406	(69.9)	0.21
Total (b)	**6.074**	**(100.0)**	**3.441**	**(100.0)**	
Admission to ICU	1.652	(27.1)	463	(13.4)	0.24
Long hospital stay (>7 days)	1.751	(28.7)	1.117	(32.4)	0.64
Referred to another place	46	(0.8)	32	(0.9)	0.68
Total	**6.103**	**(100.0)**	**3.452**	**(100.0)**	
Any *near miss* criteria on admission	196	(35.0)	95	(27.1)	0.28
Total	** 560**	**(100.0)**	** 350**	**(100.0)**	
Management indicators					
Use of magnesium sulphate	2.932	(48.0)	1.685	(48.8)	0.96
Blood product transfusion	855	(14.0)	711	(20.6)	0.13
Central venous access	261	(4.3)	102	(3.0)	0.45
Intubation unrelated to anesthesia	190	(3.1)	106	(3.1)	0.97
Hysterectomy due to infection or hemorrhage	116	(1.9)	55	(1.6)	0.69
Total	**6.103**	**(100.0)**	**3.452**	** (100.0)**	

Missing information for: (a) 564 cases; (b) 40 cases.

^1^ *P* value adjusted for cluster effect.

**Table 6:** Distribution of cases of severe maternal morbidity according to occurrence of delays in obtaining obstetric care and level of performance of care.

Type of delay, related to:	Level of performance				*P* ^1^
	Adequate		Nonadequate		
	*n*	(%)	*n*	(%)	
(1) User factors	567	(10.0)	286	(10.5)	0.89
Delay in seeking health services	303	(5.4)	139	(5.1)	0.92
Refusal of treatment	276	(4.9)	150	(5.5)	0.66
Unsafe abortion	48	(0.9)	3	(0.1)	*<0.001 *
Total	**5.645**		**2.735**		
(2) Health service accessibility	1.945	(34.4)	961	(35.0)	0.93
Total	**5.647**		**2.744**		
Difficulty accessing antenatal care	68	(1.1)	58	(2.1)	0.42
Difficulties with transportation city/hospital	54	(0.9)	63	(2.2)	0.06
Total	**6.029**		**2.819**		
Absent/inadequate antenatal care	1.909	(33.8)	783	(28.6)	0.19
Geographic difficulty in accessing health service	22	(0.4)	176	(6.4)	*<0.001 *
Total	**5.645**		**2.735**		
(3) Quality of medical care	1.046	(17.3)	1.263	(42.6)	*0.01 *
Total	**6.039**		** 2.962**		
Absence of blood products	20	(0.3)	37	(1.3)	*0.01 *
Lack of medication	67	(1.1)	50	(1.8)	0.38
Difficulty in communication between hospital and regulatory center	250	(4.1)	529	(18.8)	*0.004 *
Lack of trained staff	127	(2.1)	144	(5.1)	0.09
Difficulty in monitoring	180	(3.0)	229	(8.1)	0.18
Total	**6.029**		**2.819**		
Delay in referral/transfer of the case	143	(2.4)	149	(5.2)	0.07
Delay in diagnosis	327	(5.4)	160	(5.6)	0.96
Delay in starting treatment	416	(6.9)	186	(6.5)	0.93
Improper management of the case	589	(9.7)	629	(21.8)	0.13
Total	**6.052**		**2.879**		
Any delay	2.750	(48.3)	1.937	(64.2)	*0.04 *
Total	**5.698**		**3.018**		

^1^ *P* value adjusted for cluster effect.

**Table 7:** Variables independently associated with adequate performance (Poisson multiple analysis^1^ *n* = 9555).

Variable	PR	95% CI PR	*P* ^1^
Use of magnesium sulfate	1.44	1.04–1.98	0.03
Geographic region (SE, S)	2.21	1.05–4.65	0.04

^1^ *P* value adjusted for cluster effect; PR: prevalence ratio; SE: Southeast; S: South.
